# Ultrasound-guided femoral venipuncture for catheter ablation of atrial fibrillation

**DOI:** 10.1007/s10840-024-01918-4

**Published:** 2024-09-11

**Authors:** Jana Hašková, Josef Kautzner, Petr Peichl, Predrag Stojadinovič, Bashar Aldhoon, Peter Štiavnický, Eva Borišincová, Jiří Plášek, Robert Čihák, Dan Wichterle

**Affiliations:** 1 Department of Cardiology, Institute for Clinical and Experimental Medicine (IKEM), Vídeňská 1958/9, Prague 4, 140 21 Czech Republic; 2 Department of Medicine I, Palacky University Hospital, Olomouc, Czech Republic; 3 Centre for Research in Internal Medicine and Cardiovascular Diseases, The University of Ostrava, Ostrava, Czech Republic

The most frequent complications of catheter ablation for atrial fibrillation (AF) are related to vascular access [1, 2]. Even though they are considered relatively benign, the associated costs could be considerable [3]. Ultrasound-guided venipuncture (USGV) of femoral veins has the potential to improve the safety of the procedure [4–7].

In this retrospective study, we evaluated rates of major vascular complications (MVC) in the USGV cohort compared to historical controls (CTRL). A specific institutional tracking system for the detection of MVC was employed, which identifies all complications up to the 3-month follow-up. MVCs were defined as those requiring intervention, resulting in hematoma/bleeding (hemoglobin drop > 30 g/l), prolonging hospitalization, or resulting in rehospitalization. All patients underwent a strict protocol of groin evaluation on the day after the procedure.

Anatomical landmark-guided femoral venipuncture (CTRL group) was used from January 2010 to December 2014. USGV was used systematically (USGV group) from January 2017 to December 2020. Different fellows established venous access in 78% of cases, while experienced staff members performed the rest. In 90.6% of all procedures, the setup of vascular access was uniform — two separate left-sided (7 Fr and 11 Fr sheaths) and two right-sided (two long 8.5 F sheaths) punctures. Altogether, 5% of procedures in the early period used one right-sided 12–14 F sheath besides two left-sided (7 F and 11 F sheaths). Between 2017 and 2020, right-sided 12 Fr transseptal sheath was used in 4.4% of patients. In the early period (2010–1014), the sheaths were withdrawn after the procedure when the ACT level dropped to 170 s and compression was applied. Since 2014, the sheaths have been removed in the EP lab, and venous hemostasis was achieved by the Z stitch (altogether, 64.3% of cases).

In the initial period (2010–2012), warfarin was temporarily discontinued and bridged with weight-adjusted low molecular weight heparin (LMWH) — a total of 26.4% of cases. Between 2013 and 2020, uninterrupted OAC or minimally (single dose) interrupted NOACs were used. After the procedure, either OAC/NOAC was restarted after the drop of the ACT under 170 s — a total of 73.6% of cases.

Continuous variables were expressed as means ± standard deviation and compared by two-sided *t*-test or Mann–Whitney *U* test, as appropriate. The chi-square test with Yates correction was used for categorical variables. Predictors of MVC were examined using univariable and multivariable linear regression analysis using the step-wise forward method.

The study cohort included 4646 AF ablation procedures (2316 and 2330 cases in USGV and CTRL groups, respectively). USGV patients were older (62.3 ± 10.4 vs 60.1 ± 9.7 years, P 0.00001) and had more comorbidities as reflected by a higher CHA_2_DS_2_-VASc score (2.13 ± 1.48 vs 1.78 ± 1.35, *P* < 0.00001). The procedure duration was significantly shorter in USGV procedures than in historical controls (172 ± 55 vs 224 ± 69 min, *P* < 0.00001). The rates of MVC overall and MVC requiring surgery are shown in Fig. 1.

**Fig. 1:**
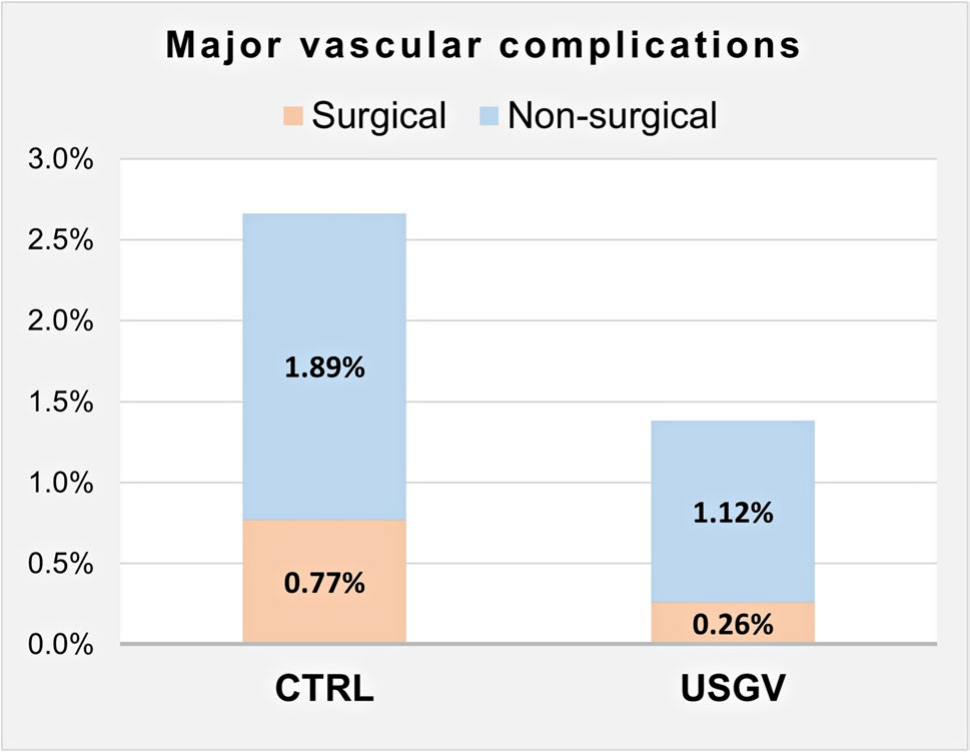
Surgical and non-surgical major vascular complications in both study groups. Abbreviations: CTRL, control group; USGV, ultrasound-guided venipuncture group

Multivariate analysis revealed that USGV strategy (*P* < 0.02), less advanced age (per a decade) (*P* < 0.006), and higher body height (per 10 cm increase) (*P* < 0.0006) were significantly associated with lower MVC rates. USGV (*P* < 0.02) and male gender (*P* < 0.03) were associated with less MVC requiring surgery. In a more detailed analysis, none of the following parameters was associated with MVC: large bore sheaths (12–14 F), discontinued warfarin with low-molecular-weight heparin bridging, uninterrupted warfarin versus one skipped dose of NOAC, and Z stitch for hemostasis. When hematoma (as a less severe complication) was not considered in the analysis, the rate of other complications such as pseudoaneurysm or AV fistula, dropped to 0.8%.

This analysis is in line with previous studies [4–7] showing the advantage of USGV. In our earlier randomized trial (4), also intra-procedural measures favored the USGV approach (puncture time 288 vs 3689 s, *P* < 0.001, first pass success 74 vs 20%, *P* < 0.001, extra-puncture attempts 0.5 vs 2.1, *P* < 0.001, inadvertent arterial puncture 0.07 vs 0.25, *P* < 0.001).

In conclusion, our retrospective analysis of two large cohorts of AF cases undergoing catheter ablation with identical introducer setups showed that USGV was associated with a statistically significant reduction of MVC. More importantly, this strategy significantly decreased the need for surgical correction. Taking together our data with other studies on this topic, ultrasound guidance of vascular access should become a standard of care in all patients undergoing catheter ablation for AF.

## Data Availability

Raw data were generated at the Institute for Clinical and Experimental Medicine, retrieved from the specific complication tracking system and placed in a purpose-built digital database. Derived data supporting the findings of this study are available from the corresponding author JK on request.

## Declarations

Josef Kautzner reports personal fees from Biosense Webster, Boston Scientific, GE Healthcare, Medtronic, and St. Jude Medical (Abbott) for participation in scientific advisory boards and has received speaker honoraria from Biosense Webster, Biotronik, Boston Scientific, Medtronic, ProMed CS, St. Jude Medical (Abbott) and Viatris. Petr Peichl has received speaker honoraria from St. Jude Medical (Abbott) and has served as a consultant for Biotronik and Boston Scientific. The remaining authors have no disclosures.
